# 267. Small colony variants causing Prosthetic joint infection depict a different gene expression profile

**DOI:** 10.1093/ofid/ofad500.339

**Published:** 2023-11-27

**Authors:** Diana Fernández-Rodríguez, José Alberto Carlos-Escalante, Luis Esaú López-Jácome, Claudia Adriana Colin-Castro, Melissa Hernández-Durán, Rodolfo García-Contreras, Toshinari Maeda, Gabriela Delgado, María del Rosario Morales-Espinosa, Rafael Franco-Cendejas

**Affiliations:** MD/PhD Plan de Estudios Combinados en Medicina (PECEM), Mexico City, Distrito Federal, Mexico; Plan de Estudios Combinados en Medicina (PECEM) MD/PhD, Mexico City, Distrito Federal, Mexico; Instituto Nacional de Rehabilitación "Luis Guillermo Ibarra Ibarra", Mexico City, Distrito Federal, Mexico; Instituto Nacional de Rehabilitación "Luis Guillermo Ibarra Ibarra", Mexico City, Distrito Federal, Mexico; Instituto Nacional de Rehabilitación "Luis Guillermo Ibarra Ibarra", Mexico City, Distrito Federal, Mexico; Universidad Nacional Autónoma de México, Mexico City, Distrito Federal, Mexico; Kyushu Institute of Technology, Kitakyushu, Fukuoka, Japan; Universidad Nacional Autónoma de México, Mexico City, Distrito Federal, Mexico; Universidad Nacional Autónoma de México, Mexico City, Distrito Federal, Mexico; Instituto Nacional de Rehabilitación "Luis Guillermo Ibarra Ibarra", Mexico City, Distrito Federal, Mexico

## Abstract

**Background:**

Prosthetic joint infection (PJI) is an infectious complication after total joint arthroplasty (TJA) with a high socioeconomic impact. Staphylococcus species attain for more than half of PJI cases. Biofilm formation and the development of small colony variants (SCV) have proven to enhance chronic and/or relapsing bacterial infections. In this regard, a better understanding of these factors can help overcome PJI and other device-associated infections. Thus, we aimed to analyze the clinical and microbiological differences between common/wild type (WT) and SCV Staphylococcus epidermidis strains

**Methods:**

We analyzed a monomicrobial cohort of PJI patients affected by *S. epidermidis*. The bacterial isolates of patients with more than one-year of follow-up were examined for SCV detection. Then, we determined the genetic relatedness between strains with a pulsed field gel electrophoresis (PFGE). Finally, we selected 4 representative strains (2 WT and 2 SCV strains) to perform a differential expression analysis by RNA-seq. We performed a multiple test correction by controlling the false discovery rate (FDR) at 10%.

**Results:**

*S. epidermidis* SCV affected 16 (37.6%) patients with monomicrobial PJI. The DNA fingerprints showed a high similarity, according to the Dice coefficient, between SCV strains and their WT counterparts. Nevertheless, further experiments demonstrated 22 genes with a significant differential expression profile: 12 were associated to metabolic pathways (carbon, amino acids, nucleotide, purines) and biosynthesis of secondary metabolites and cofactors, 3 with transmembrane transportation, 2 with redox balance, 1 with pH balance, 1 with integral components of the membrane and cell adhesion. Function was not identified for 3 of these genes.

Differential gene expression profile among SCV isolates causing prosthetic joint infection.

**Figure:**
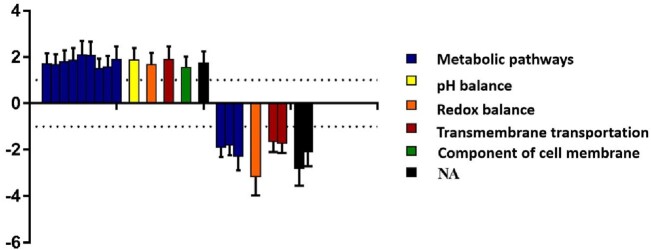

**Conclusion:**

SCV are common in device-related infections like PJI. *S. epidermidis* SCV and their WT counterparts showed an extremely similar genetic background: still, SCV strains have a different gene expression profile. These findings explain some phenotypic variations (growth rate, biofilm properties, antimicrobial susceptibility profiles) found in clinical SCV strains. These genes may be promising therapeutic targets for combating chronic and/or relapsing bacterial infections.

**Disclosures:**

**All Authors**: No reported disclosures

